# Crowdsourcing: It Matters Who the Crowd Are. The Impacts of between Group Variations in Recording Land Cover

**DOI:** 10.1371/journal.pone.0158329

**Published:** 2016-07-26

**Authors:** Alexis Comber, Peter Mooney, Ross S. Purves, Duccio Rocchini, Ariane Walz

**Affiliations:** 1 School of Geography, University of Leeds, Leeds, United Kingdom; 2 Department of Computer Science, National University of Ireland Maynooth, Ireland; 3 Department of Geography, University of Zurich, Zurich, Switzerland; 4 Fondazione Edmund Mach, San Michele all'Adige, Italy; 5 Institute of Earth and Environmental Science, University of Potsdam, Potsdam, Germany; University of Missouri, UNITED STATES

## Abstract

Volunteered geographical information (VGI) and citizen science have become important sources data for much scientific research. In the domain of land cover, crowdsourcing can provide a high temporal resolution data to support different analyses of landscape processes. However, the scientists may have little control over what gets recorded by the crowd, providing a potential source of error and uncertainty. This study compared analyses of crowdsourced land cover data that were contributed by different groups, based on nationality (labelled Gondor and Non-Gondor) and on domain experience (labelled Expert and Non-Expert). The analyses used a geographically weighted model to generate maps of land cover and compared the maps generated by the different groups. The results highlight the differences between the maps how specific land cover classes were under- and over-estimated. As crowdsourced data and citizen science are increasingly used to replace data collected under the designed experiment, this paper highlights the importance of considering between group variations and their impacts on the results of analyses. Critically, differences in the way that landscape features are conceptualised by different groups of contributors need to be considered when using crowdsourced data in formal scientific analyses. The discussion considers the potential for variation in crowdsourced data, the relativist nature of land cover and suggests a number of areas for future research. The key finding is that the *veracity* of citizen science data is not the critical issue per se. Rather, it is important to consider the impacts of differences in the semantics, affordances and functions associated with landscape features held by different groups of crowdsourced data contributors.

## Introduction

The scientific community in general is excited by the opportunities afforded by the related fields of crowdsourcing, volunteered geographical information and citizen science. There has been an explosion of applications underpinned by crowdsourced data in many areas of scientific investigation: from astronomy [1] to zoology [2]. One key attraction of such data relates to high data volumes for relatively low costs. In the domain of land cover and land use, the European Commission has funded a number of projects to evaluate how volunteered or crowdsourced data may be used to help manage crises and emergencies [3], to develop Citizen Observatories for Land Cover and Land Use [4] and to monitor deforestation [5]. The reasons for these initiatives in the context of land cover are various but include the potential financial benefits of using crowdsourced data and the advantages of involving citizens more directly in science. Land cover data collection is expensive: sampling for the LUCAS project [6] cost €6.42m. As a result a number of crowd-sourced land cover data collection systems have been initiated with perhaps the best known of these being the Geo-Wiki system developed at IIASA, Austria [7] although others exist [8, 9, 10]. Geo-Wiki has been used for a number of campaigns [11] and has seen considerable refinement in interfaces and platforms, the campaigns it has run and in the applications it supports, as well as increased data volumes and contributor numbers. The basic premise of Geo-Wiki is to produce open data by allowing citizens to either provide feedback on existing data or create entirely new data [12].

Whilst a considerable range of work has considered data quality issues related to the veracity of Geo-Wiki land cover data [11, 13, 14], as yet little work has examined the impacts of variations in the data contributed by different groups which may reflect divergent landscape conceptualisations.

This paper considerably extends and refines initial work reported in [15]. It evaluates the impacts on decision making of variations what gets recorded by contributors from different countries and with different levels of expertise. It compares inferences about the presence and spatial distribution of land cover by analysing crowdsourced land cover data contributed by two sets of groups. The first compared data contributed by volunteers from one country, named *Gondor* to avoid making inferences based on national stereotypes, and data from all other nationalities. The second compares data contributed by experts with non-experts. The analyses show how data contributed by different groups of people result in different inferences and highlight the potential impacts of unintended (and unknown) variations in crowdsourced data.

## Background

There are many citizen science and crowdsourced data generation activities, which in the realm of geographical information science and systems is frequently referred to as ‘VGI’ (*volunteered geographical information*), a phrase coined by Goodchild [16], and recent developments are reviewed in [17]. Many contributors provide data for free because of their interest in a particular topic, although sometimes in return for some token reward through gameification [18] or electronic money [19]. Concerns over the use of crowdsourced spatial data in formal scientific analyses remain because of data quality issues [13]. Data quality in this context encompasses a number of considerations, which, in increasing complexity, relate to:

- Veracity and error. Is the crowdsourced datum correct? Is the land cover present at a given location correctly identified or labelled?
- Sampling and stratification. Do the data adequately capture the variation in the process under investigation, in extent as well as spatially and thematically? Is the density of data points of different classes sufficient to capture the spatial distribution of the land cover present on the ground?
- Observation scale and grain. Does the granularity of recording vary, both between individuals and with the intended analysis?

In traditional scientific activities, such considerations are addressed by a formal experimental design which includes activities and protocols to ensure the inferential statistical robustness of any data analysis. These may involve staff training, the application of certain measurement thresholds and instrument calibration, all of which impose control over the epistemology of data collection, as well as error checking protocols, sampling designs, and quality assurance procedures. In contrast, for geographical analyses that use crowdsourced data, the scientist has little control over the spatial distribution of the volunteer locations, has to take the veracity of observations on trust and can only assume that the perceptions of landscape features held by the crowd are appropriate for the intended analysis.

**Fig 1:**
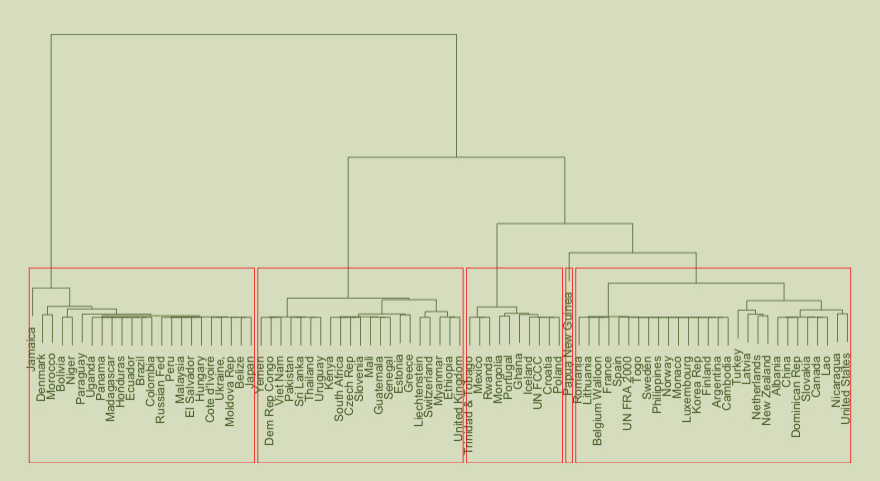
Clusters countries with similar definitions of ‘forest’ based on minimum area, tree height and canopy cover.

Consideration of how landscape feature are conceptualised is critical especially in heavily socially constructed areas of science such as land cover [20]. This is because different people, from different backgrounds and cultures or with different experiences (including disciplinary training) have been frequently found to hold different underlying conceptualisations of landscape features and categories. By way of example, consider the concept of a *forest*. A number of researchers have discussed the many ways that *forest* is conceived, from early work by Bennet [21] through to Comber et al [22]. These are illustrated by the many national definitions of forest. Gyde Lund maintains a list of forest definitions [23], with active hyperlinks. These include descriptions of the minimum physical requirements for areas of tree covered land to be considered as ‘forest’ in different countries. To illustrate this variation Fig 1 show a *k-means* classification of values for the minimum area, tree height and canopy cover for forest definitions from different countries, grouped into 5 clusters. The mean rescaled values for each cluster are shown in Table 1. Notice that the forest class definitions cluster reveals that some countries have similar definitions (Viet Nam, Pakistan, Sri Lanka, for example) and that some clusters are nearer to each other in the feature space (Table 1). Fig 1 illustrates how national concepts associated with land cover vary. It highlights an important consideration if crowdsourced data are to be used in scientific analyses: the potential for variation in the way that similar landscape features may be labelled by different groups of crowdsourced data contributors with the result that different groups may identify different features as being present at the same location.

**Table 1:** The mean rescaled values of the 5 clusters of forest definitions.

Cluster	Area	Cover	Height
1	-0.110	-1.100	0.691
2	-0.135	0.536	-1.112
3	-0.084	0.611	0.698
4	8.976	-1.100	0.617
5	-0.138	-1.052	-1.274

The importance of considering the potential for such variations is because crowdsourcing data such as Geo-Wiki are now within mainstream scientific investigation. Geo-Wiki uses an interface with Google Earth imagery to collect volunteered land cover data under a nominal pixel of 250m to 500m depending on the campaign. The data has been used to assess the quality of existing land cover products [24], to quantify their uncertainties [25] and to generate hybrid global land cover maps [26]. Some work has examined the sources of variation in Geo-Wiki data and initial studies found differences between experts and non-experts in identifying land cover, with experts more accurate than non-experts in class allocation [11]. This was extended to examine the impacts of expertise on decision making, which were found to be more profound in some continents than in others [27]. Other work has examined Geo-Wiki data quality by comparing them with control points [13, 11] and through latency analyses [14, 28]. Little work has directly considered the impacts of conceptual variations, linguistic or cultural factors [29, 30] in Geo-Wiki data that, in other work have been found to result in large differences in the ways that landscape features are conceptualised [31]. Such *ethnophysiographic* differences [32, 33] are well known in the context of formal land cover creation [34] and landscape analyses [29, 35]. As yet no work has considered the potential impacts of variations in how landscape features are conceived amongst different groups of Geo-Wiki contributors.

## Methods

An analysis of Geo-Wiki data was used to infer the land cover at each location on a 50km grid covering North and South America, with 22,730 cells in total. The approach was to use a moving window or kernel to extract Geo-Wiki data near to each location. The data were weighted by their distance to the kernel centre and a geographically weighted regression was then used to infer the land cover at each location. The land cover type with the highest coefficient estimate was used allocated as the class at that location.

### Data and Case study

**Fig 2:**
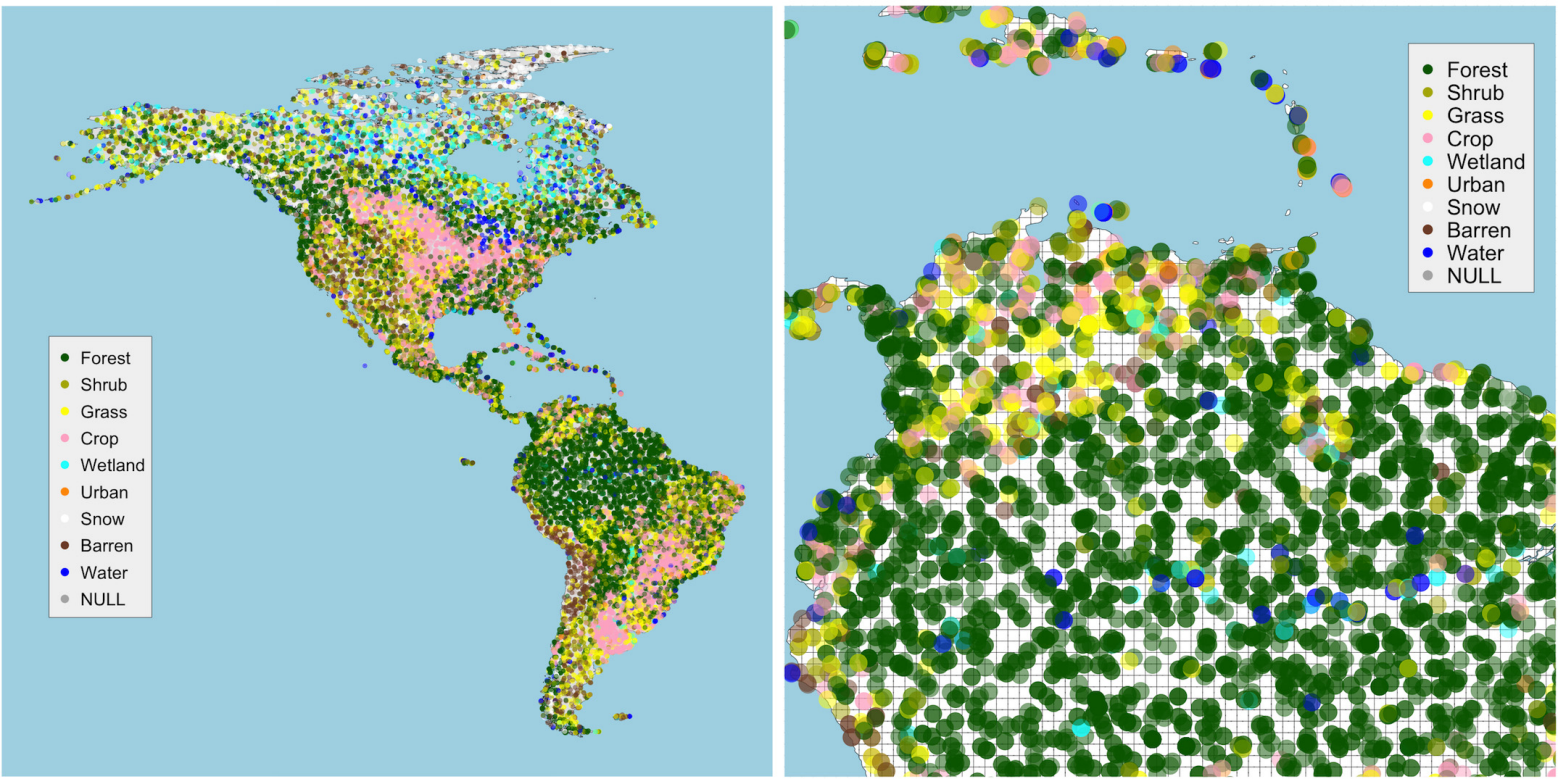
The distribution of the data a) in the case study area and b) local detail showing the density of the data points shaded with a transparency term and the 50km analysis grid.

The analysis used data collected through the Geo-Wiki project [7, 36]. It has web and smartphone app interfaces and is open to anyone. There have been different campaigns targeted at specific land related processes and phenomenon, including bio-fuels, forest biomass and more recently livestock distributions. As part of the Geo-Wiki registration process, volunteers are asked to describe their level of expertise and the country they are from. Once registered, volunteers can contribute to different campaigns in which they allocate what they observe from Google Earth imagery at a series of randomly selected locations, to one of a predefined set of classes. ***NB*** The Geo-Wiki classification has 10 classes but the *Mosaic* class was excluded from this analysis because of its inherent ambiguity. Instructions explain the operation of Geo-Wiki but little detail is provided about the land cover classes. In this research, data from three Geo-Wiki campaigns were combined. One dataset contained data from contributors from one country (*‘Gondor’*), the other two contained data from a mix of contributors mostly of other nationalities, but with some from Gondor. They were chosen because each campaign had similar objectives. These were combined and a subset of data covering North and South America was extracted. The selection of this study area was simply to provide a case study whose landscapes are familiar, with a broad sequence of arctic, tundra, grass plains, desert areas, tropical forest, grass plains running from North to South. The distributions of the data amongst the classes for Gondor and Non-Gondor and Expert / Non-Expert, with combinations thereof in the study area, are summarised in Table 2. Of the contributors, 20 were from *Gondor*, 119 people were of other nationalities, 76 declared themselves to be experts in land cover and remote sensing and 64 as Non-Experts. The 30,303 points in the study area are shown in Fig 2. The combinations are included for illustrative purposes only–the differences in the number of data points contributed for example by Gondor-Expert and Non-Gondor-Non-Expert are too few to develop any meaningful spatial comparisons.

**Table 2:** The distributions of the land cover data points collected by contributors with different backgrounds.

Class	Total	Non-Gondor	Gondor	Non-Expert	Expert	Non-Gondor Expert	Gondor Expert	Non-Gondor Non-Expert	Gondor Non-Expert
Forest	10754	33	40.2	38.4	32.5	32.6	31.8	33.7	42.6
Shrub	3407	7.7	18.1	10.7	11.8	8.1	33	7.1	14
Grass	4925	16.9	15.1	16.7	15.8	16.5	11.6	17.4	16.1
Crop	5334	20.8	11.4	16.3	18.9	20.1	12.6	22	11.1
Wetland	1325	5.4	2.4	3.6	5.2	5.7	2.3	4.9	2.4
Urban	331	1.1	1	1	1.2	1.2	1.3	1	1
Snow	969	3.6	2.5	2.8	3.6	4.1	1.2	2.7	2.8
Barren	2193	8	5.8	7.1	7.4	8.2	3.1	7.7	6.5
Water	1065	3.5	3.5	3.5	3.6	3.6	3.2	3.3	3.6
Total	30303	20004	10299	15445	14858	12643	2215	7361	8084

**Fig 3:**
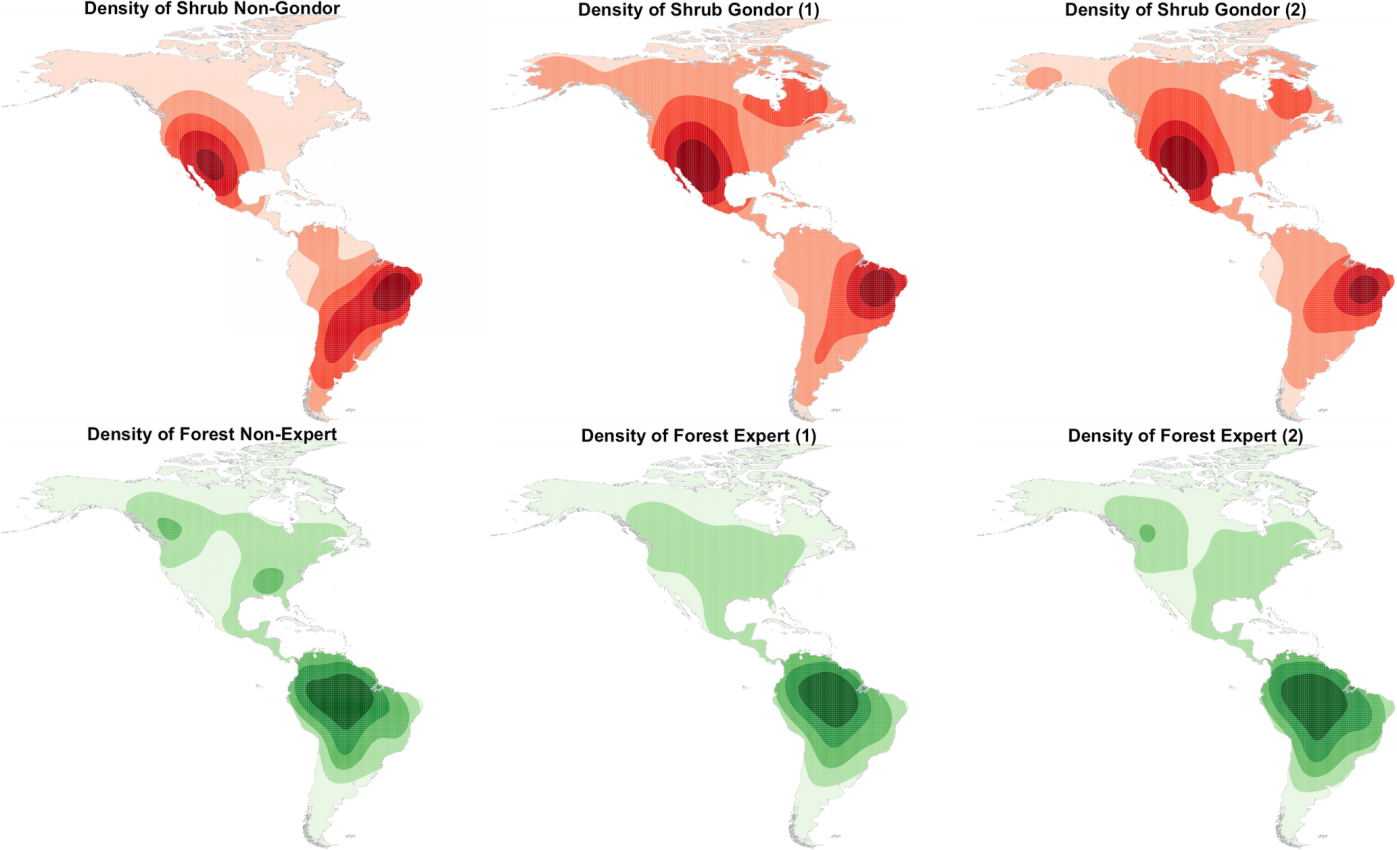
Kernel Density Estimation surfaces of the distributions of a) *Shrub* cover comparing Gondor and Non-Gondor groups and b) *Forest* cover comparing Expert and Non-Expert groups. Darker areas indicate a greater density of data points and in both cases the Expert and Gondor groups were randomly split and mapped.

It is evident from Table 2 that despite a random sample of locations, for some classes there are large differences of the number of points allocated to each class by Gondor and Non-Gondor groups, with less difference between Expert and Non-Expert groups. For example, there are large differences in the number of locations classified as *Shrub* by Gondor and Non-Gondor and as *Forest* by Experts and Non-Experts. In contrast there is a much greater degree of homogeneity in the identification of *Grass* and *Crop* classes. The spatial implications of these differences can be visualised using a Kernel Density Estimation. The KDE bandwidth was derived automatically from the heuristic suggested by Venables and Ripley ([37], p127) and implemented in the *bandwidth*.*nrd* function included in the *MASS* package for R, the open source statistical software. The KDE surfaces arising from different groups are shown in Fig 3. It illustrates the differences in the spatial distributions of *Shrub* data between Gondor and Non-Gondor and *Forest* between Experts and Non-Expert. The general distributions of these classes are similar–that is they have the same broad regions of different land covers–but with interesting and potentially important local variations. It is possible that the mapped differences may be simply due to different data point locations which some a sensitivity analysis could quantify, but the locations were randomly sampled (Fig 2). In this case, when a group (i.e Gondor or Expert) is randomly split into two subsets and KDEs are generated from that data, the models are similar (Fig 3).

### Analysis

A Geographically Weighted Regression (GWR) [38, 39] was used to infer the land cover at each location in a 50km grid from the Geo-Wiki data subsets. A geographically weighted kernel generated geographically weighted averages for each class, at each grid location under the assumption that the land cover at any given location can be determined by examining values at nearby locations (i.e. that land cover exhibits spatial autocorrelation). The class with the greatest coefficient was inferred as the class at that location. Then the land cover maps from Gondor vs. Non-Gondor data and Expert vs. Non-Expert data were compared.

Geographically Weighted (GW) models have been used in many Geo-Wiki applications as they provide a framework for integrating and analysing data that accommodates the well-known spatial autocorrelation of many landscape processes and features [40, 41]. In the context of Geo-Wiki, Lesiv et al [42] used a GW framework to create a hybrid forest map, Comber et al [13] used geographically weighted kernels to generate local measures of Geo-Wiki accuracy and Schepaschenko et al [43, 44] used a GW regression approach to integrate different data related to forestry. Comber et al [45] evaluated the GW approach against other models of spatial inference, belief and evidence combination and found the GW framework to produce the most accurate results.

In an ideal experimental design a large number of classifications of the same area by different groups would be compared against some reference data. Theoretically one or some of the many global land cover datasets could have been used to do this. However, there are many well-recognised and long-standing problems when using any of the global land cover datasets as a referent: they have different nomenclatures, spatial scales and thematic granularities and as a result describe the world in very different ways [46]. They have profound disagreements of the amount and distribution of land covers as documented by numerous authors, [26, 47, 48], they do not correspond to official land cover statistics at national or regional levels [49]. Indeed global land cover datasets are so unreliable that Geo-Wiki has been used to determine which of them best describes the land cover in different places in order to suggest a the composition of hybrid dataset [26]. For these reasons, this analysis sought to identify the nature and direction of any differences in the land cover generated using a GW averaging approach, rather than to compare the class labels with a referent.

GWR is similar to an ordinary regression but computes a series of local regressions. A moving window or kernel is passed over the study area. Data under the kernel are weighted by their distance to the kernel centre and then used to calibrate a local regression model. In this way the outputs of GWR allow regression coefficients to vary spatially compared to a single global coefficient estimate using standard regression. In this analysis local, GW models were computed over a grid of locations spaced at 50km, a portion of which is shown in Fig 2. The shape and size of the GW kernel affect the degree of smoothing [50]. Here data points were weighted using a tri-cube function with a 50km bandwidth. This bandwidth reflected an acceptable degree of spatial aggregation and the tri-cube shape provided an appropriate distance weighting function. The distribution of the Geo-Wiki data points under the kernel was as follows: 1,547 of the 50km cells had no data; of the 28,756 cells that did contain Geo-Wiki data, the 1^st^ quartile, median and 3^rd^ quartile were 1, 2 and data points respectively.

The GWR model was parameterised to compute geographically weighted means (*y*) for each class *c* at each location *i* as follows:

$$y_{c_{\left(u_{i},v_{i}\right)}}=\beta_{0_{\left(u_{i},v_{i}\right)}}$$ (1)

where (*u*_*i*_, *v*_*i*_) is a vector of two dimensional co-ordinates describing the location of *i* over which the coefficient estimates are assumed to vary. In this way the outputs provide a geographically weighted mean estimate with a value in the range [0, 1] at each grid location, for each class. The class with the highest value was used to label the location. This is a smoothing approach similar to that used by Comber et al (2012) to determine fuzzy accuracy distributions.

**Fig 4:**
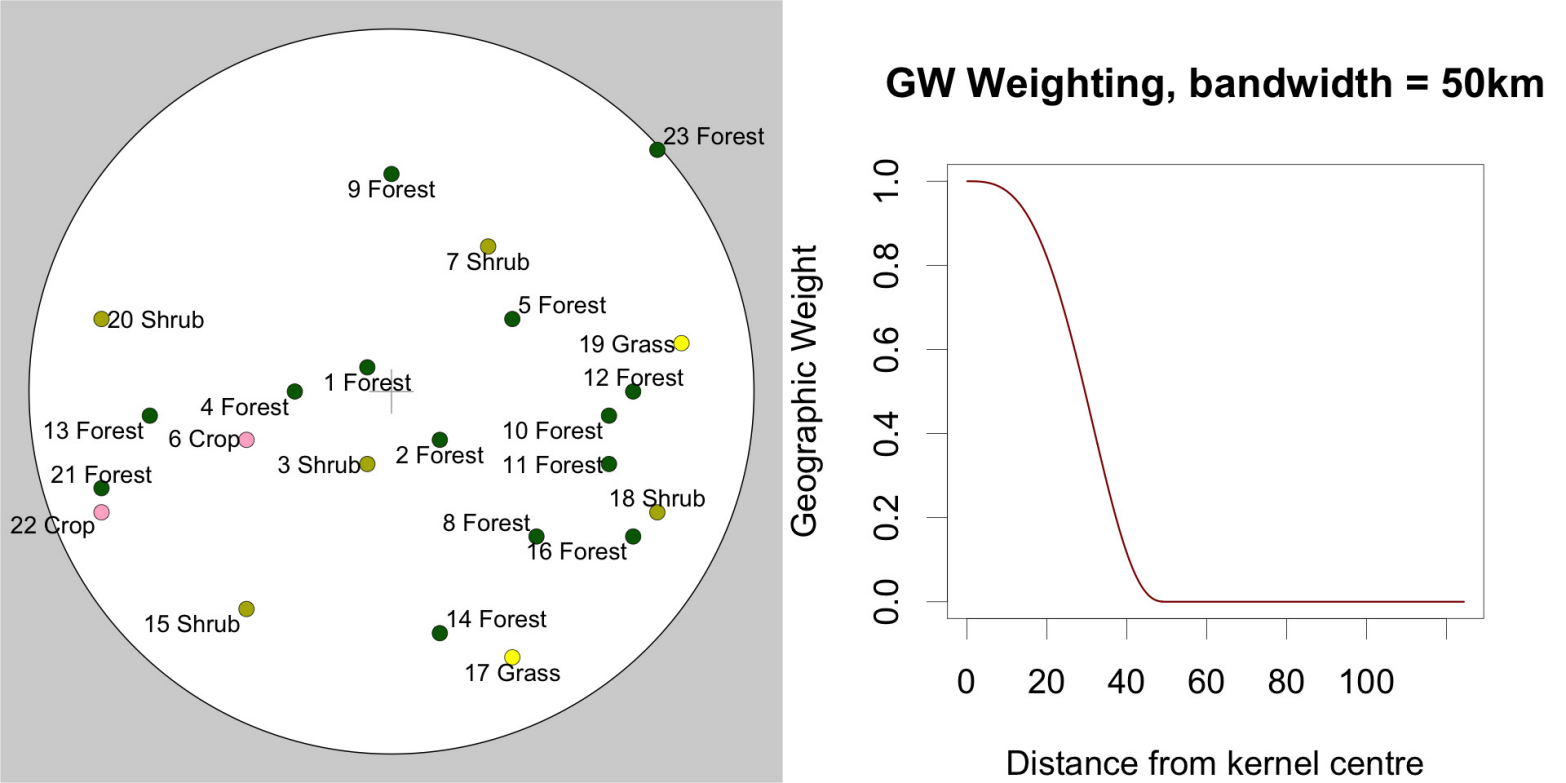
a) An example of Geo-Wiki crowdsourced data points within a 50km buffer under a kernel centred on coordinates (0,0) and b) the weighting function used in the geographically weighted average approach.

To illustrate the operation of the GW kernel, Table 3 shows some hypothetical Geo-Wiki data, with distances to the sample point (kernel centre, grid cell location) being considered and the weights derived from the tri-cube function. Fig 4A shows data centred around the sample point within a 50km buffer. Fig 4B shows how the tri-cube function generates weights for each data point based on their distance to the kernel centre.

**Table 3:** The hypothetical Geo-Wiki data used to exemplify the geographical weighting method used as inputs to the geographically weighted average.

ID	Land Cover Class	X	Y	Distance (km)	Geographic Weight
1	Forest	-3	3	4.7	0.997
2	Forest	7	-7	9.4	0.980
3	Shrub	-3	-10	10.5	0.972
4	Forest	-13	0	13.3	0.944
5	Forest	17	10	19.4	0.834
6	Crop	-20	-7	21.1	0.792
7	Shrub	13	20	24.0	0.702
8	Forest	20	-20	28.3	0.549
9	Forest	0	30	30.0	0.482
10	Forest	30	-3	30.2	0.475
11	Forest	30	-10	31.6	0.417
12	Forest	33	0	33.3	0.348
13	Forest	-33	-3	33.5	0.342
14	Forest	7	-33	34.0	0.322
15	Shrub	-20	-30	36.1	0.244
16	Forest	33	-20	38.9	0.149
17	Grass	17	-37	40.3	0.109
18	Shrub	37	-17	40.3	0.109
19	Grass	40	7	40.6	0.102
20	Shrub	-40	10	41.2	0.085
21	Forest	-40	-13	42.2	0.064
22	Crop	-40	-17	43.3	0.043
23	Forest	37	33	49.6	0.000

The weighted data were then analysed on a class-by-class basis using Eq 1 to determine the land cover class with highest coefficient. In the example above the coefficients from the analysis are shown in Table 4 and in this case the class of ‘Forest’ would be allocated to the sample point under consideration. This method was used to generate surfaces of land cover from crowdsourced data using the whole dataset and specific subsets reflecting different expertise and nationality.

**Table 4:** Coefficient estimates arising from the geographically weighted regression example.

Forest	Shrub	Crop	Grass
0.300	0.092	0.036	0.009

## Results

**Fig 5:**
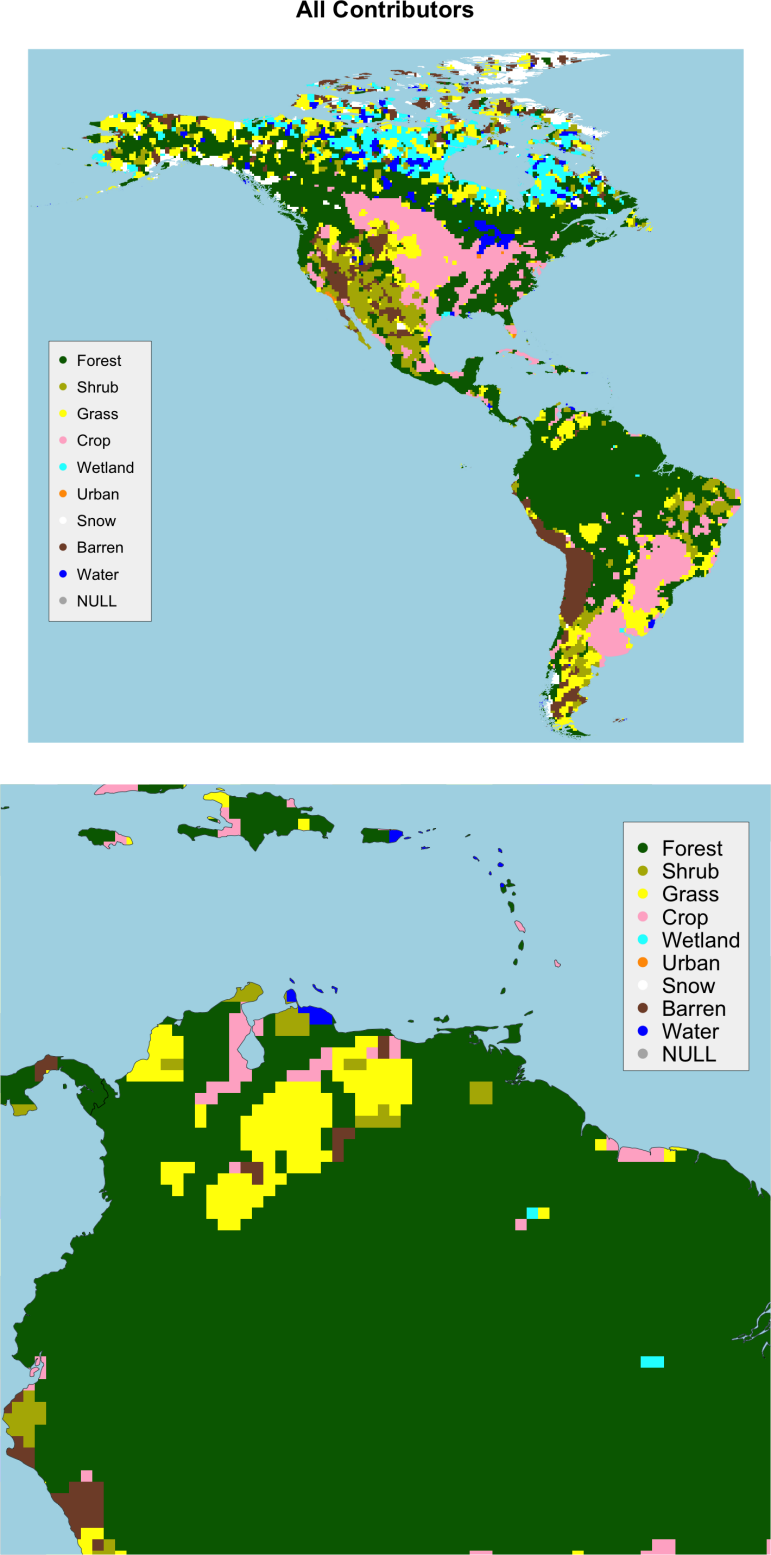
The land cover data over a 50km grid a) generated from all contributors in the study area and b) with some local detail.

The crowdsourced Geo-Wiki data for North and South America was analysed using the GW averaging approach described above to determine local means for each class, for each cell in a 50km grid. The land cover class with the highest mean was assigned as the class for each cell. Fig 5 shows the land cover map generated in this way using data from all contributors, with the same detail as in Fig 2.

### Gondor vs Non-Gondor

**Fig 6:**
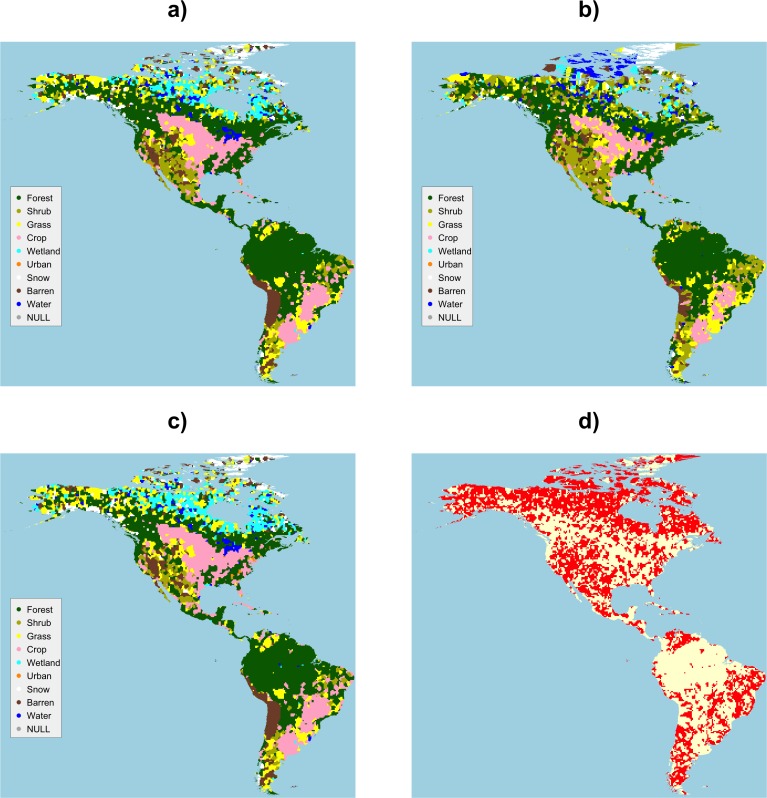
The land cover maps generated by data from a) all contributors, b) contributors from Gondor, c) Non-Gondor and d) a map of difference, with differences in red.

The maps in Fig 6 are those generated by All Contributors, by the Gondor and Non-Gondor subsets and a map of difference, showing the locations where different land cover classes were assigned from the analysis of Gondor and Non-Gondor data. A visual inspection of the maps suggests that the *Non-Gondor* map is similar to All Contributors map. The main areas of similarity between the *Gondor* map and the All Contributors map are the agricultural areas (labelled as *Crop*) in the great plains of North America and in the Pampas lowlands in South America, and the *Forest* areas in Amazonia. Interesting and potentially significant differences are the subtle but important differences in the distributions of the *Wetland* class in the north, *Shrub* and *Barren* in western North America and the Northeast Region of Brazil.

**Fig 7:**
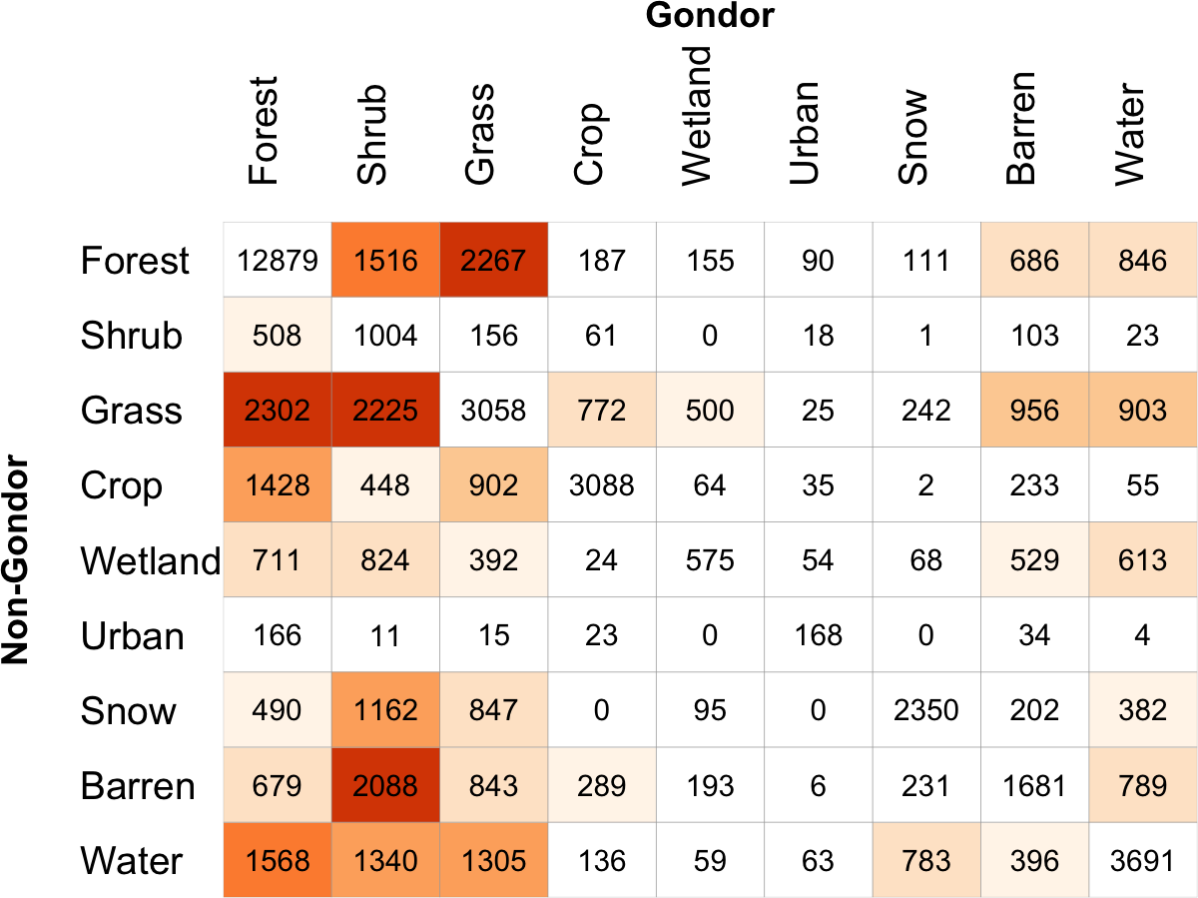
The correspondence matrix of the land cover maps generated from data contributed by Gondor and Non-Gondor subsets. Diagonal agreement and low levels of off-diagonal disagreement are indicated in white, with increasing levels of off-diagonal disagreement shaded from light to dark orange.

The origins of these differences and how they relate to specific classes can be examined through a contingency table summarising the per grid cell correspondence between the mapped datasets. A correspondence matrix allows the degree and nature of differences in the way that different groups classify land cover to be quantified, under the assumption of spatial-autocorrelation of land cover. The correspondences between the Gondor and Non-Gondor maps are shown in Fig 7. These summarise the intersection of the maps shown in Fig 6 and the shading indicates the relative off diagonal differences. Reading across the rows, the table values indicate the number of grid cells allocated to each class by each group. For example, out of the 18,737 grid cells allocated to the class of *Forest* by Non-Gondor contributors, 2,267 were given the label *Grass* by contributors from Gondor. It is evident that there are high levels of differences in the interpretation of *Grass* and *Shrub* classes between the Gondor and Non-Gondor contributors. Fig 7 suggests that contributors from Gondor differ from the general trend particularly in their allocation and interpretation of these land cover classes and the *Forest*, *Barren* and *Water* classes.

If the analysis of data from All Contributors (Fig 6A) is considered as some kind of reference dataset then the correspondence matrix can be used to construct class specific measures of Omission and Commission. These are derived from the row and column marginal totals. The full correspondence matrices comparing land cover maps derived from All Contributors against the Gondor and Non-Gondor subsets and the derived measures of Omission and Commission are included in S1 Table, S2 Table, S3 Table and S4 Table and are summarised in Table 5.

**Table 5:** Omission and Commission differences between maps generated from all data and from Gondor and Non-Gondor groups.

		Forest	Shrub	Grass	Crop	Wetland	Urban	Snow	Barren	Water
**Omission**	**Gondor**	0.20	0.10	0.43	0.39	0.70	0.43	0.41	0.63	0.39
	**Non-Gondor**	0.17	0.70	0.29	0.09	0.13	0.19	0.16	0.13	0.13
**Commission**	**Gondor**	0.23	0.59	0.41	0.22	0.43	0.46	0.19	0.49	0.37
	**Non-Gondor**	0.12	0.22	0.35	0.15	0.27	0.17	0.21	0.14	0.29

Considering first the Omissions, these indicate the proportions of each reference class that were allocated to a different class. They are calculated from 1 minus the diagonal element in the full correspondence matrix in S1 Table, S2 Table, S3 Table and S4 Table, divided by the row total. There are large differences between Gondor and Non-Gondor in *Shrub*, *Grass*, *Crop*, *Wetland*, *Urban*, *Snow*, *Barren* and *Water*. These Omission values indicate that contributors from Gondor under-estimate *Grass*, *Crop*, *Wetland*, *Urban*, *Barren*, *Snow* and *Water* when compared to all contributors and that Non-Gondor contributors under-estimate *Shrub*.

The Commissions indicate the proportions of each class derived from analysis of the group data (Gondor or Non-Gondor) that were a different class in the reference data. Commission values are calculated from 1 minus the diagonal element in the full correspondence matrix in S1 Table, S2 Table, S3 Table and S4 Table divided by the column total. There are large differences evident in the *Forest*, *Shrub*, *Wetland*, *Urban* and *Barren* classes with all of these classes over-estimated by the contributors from Gondor.

### Expert vs Non-Expert

**Fig 8:**
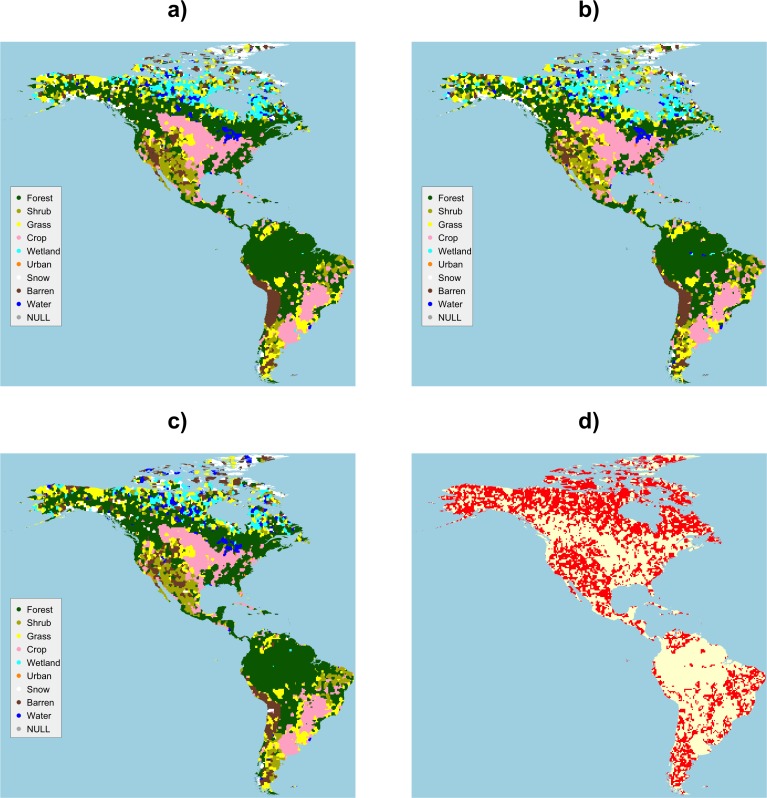
The land cover maps generated by data from a) All Contributors, b) Expert contributors, c) Non-Experts and d) a map of difference, with differences in red.

**Fig 9:**
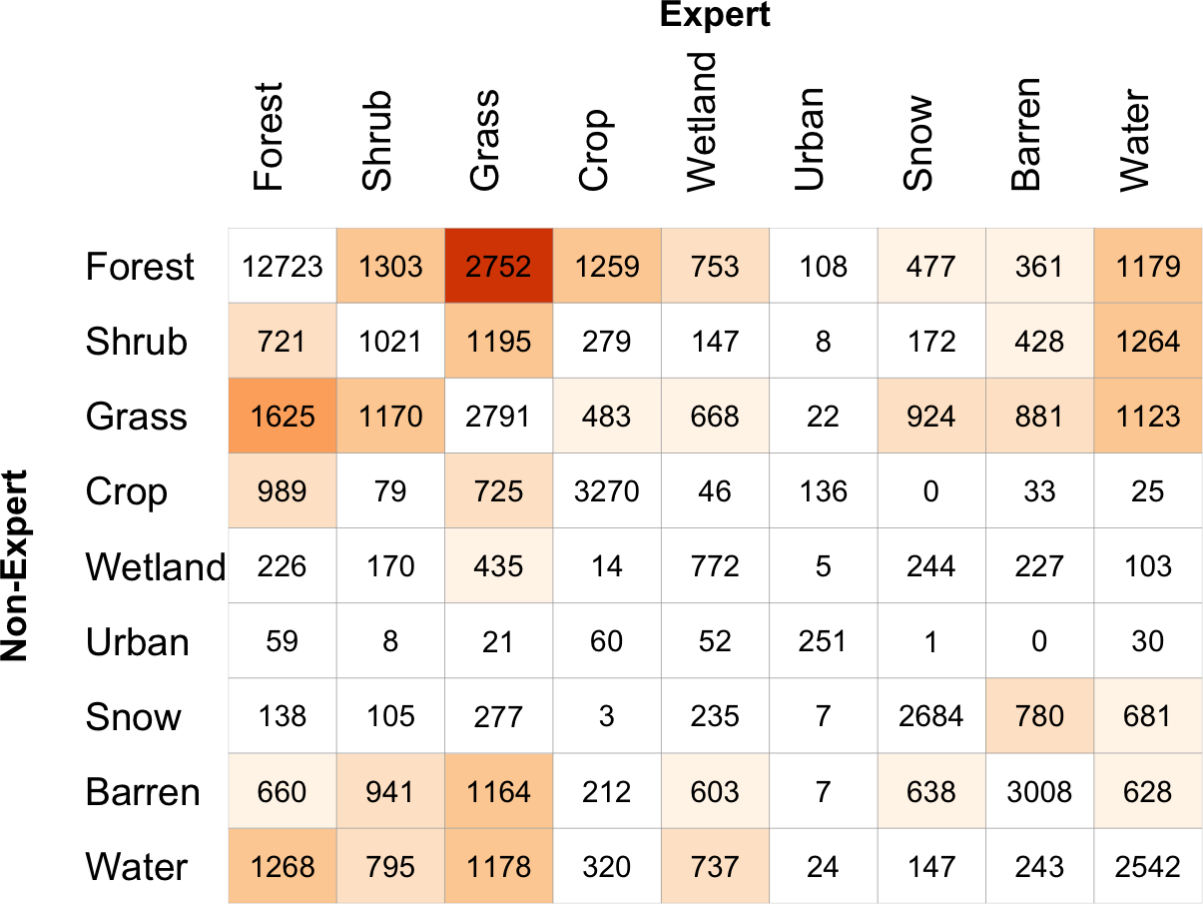
The correspondence matrix of the land cover maps generated from data contributed by Expert and Non-Expert subsets. Diagonal agreement and low levels of off-diagonal disagreement are indicated in white, with increasing levels of off-diagonal disagreement shaded from light to dark orange.

The land cover maps derived from Expert and Non-Expert data were compared in the same way. Fig 8 shows the mappings and Fig 9 shows the correspondence matrix. The map of difference in Fig 8D) and the off-diagonal values in Fig 9 indicate] that there are fewer differences between Experts and Non-Experts than between Gondor and Non-Gondor. The largest differences are in the way that *Forest* and *Grass* land covers are interpreted.

Table 6 summarises the marginal Omission and Commission rates calculated from the full correspondence matrices between All Contributors and Expert and Non-Expert groups. The large differences between Experts and Non-Expert mappings are as follows: there are greater Omissions associated with Expert mappings of *Shrub* and *Water* and with Non-Expert mappings of *Wetland*. The Commissions indicate large differences associated with the Expert mappings of *Shrub*, *Grass*, *Urban*, *Barren* and *Water*. So differences exist between Experts and Non-Experts for some classes: Experts are more likely miss (omit) *Shrub*, *Grass* and *Water* compared to all contributors and Non-Experts to include (commit) *Barren* for example.

**Table 6:** Omission and Commission differences between maps generated from all data and from Expert and Non-Expert groups.

		Forest	Shrub	Grass	Crop	Wetland	Urban	Snow	Barren	Water
**Omission**	**Expert**	0.22	0.46	0.40	0.21	0.20	0.22	0.24	0.32	0.37
	**Non-Expert**	0.15	0.35	0.35	0.24	0.60	0.22	0.25	0.26	0.28
**Commission**	**Expert**	0.16	0.53	0.43	0.22	0.36	0.40	0.25	0.22	0.38
	**Non-Expert**	0.19	0.40	0.32	0.16	0.42	0.29	0.21	0.36	0.26

## Discussion

The land cover maps indicate analyses using crowdsourced data contributed people from different national groups will vary. A comparison of Gondor and Non-Gondor land cover maps suggested large differences in the allocation of Forest, Shrub and Grass classes (Fig 7) and in the allocation of Snow, Barren and Water, although these classes were less frequent. In contrast, much less variation was found when Expert and Non-Expert groups were compared, with large differences only in the mappings of *Forest* and *Grass* land cover. The analysis used a GW model to infer a spatial distribution of land cover from Geo-Wiki data points which operates under the well-known assumption of the spatial autocorrelation of land cover. If this assumption is correct and the data sampling has no effect (see Fig 3), then the variation in land cover maps is due to differences in the labelling by different groups of contributors. Gondor interpret the landscape in different ways to those from other countries and the mapping variations reflect group conceptualisations of landscape features and processes with significant epistemological and ontological differences. Alternatively, these variations may be due to simple linguistics.

However, it is well known that different contributors, with different experiences, training and backgrounds have varying perspectives on the world, or *Weltanschauung*. Even people from the same region or with the same level of expertise will disagree about the land cover present. To illustrate this point, consider 3 of the Geo-Wiki locations and the land cover classes that were assigned to them by different groups in Tables 7, 8 and 9. These show how the same points were similarly classified by individuals in different groups (Table 7 and partially in Table 8) and how other locations are classified in very different ways (Table 9). This may be due to the inherent heterogeneity of the land cover present but it may be due to different group conceptualisations of the landscape. What is certain is that such variations can have profound implications for scientific analyses that incorporate crowdsourced data.

**Table 7:** An example of the land cover classes indicated a Geo-Wiki location, with low variation in opinion between Gondor and Non-Gondor groups.

	Non-Gondor	Gondor
Grass	1	1
Crop	1	0
Barren	24	18

**Table 8:** An example of the land cover classes indicated a Geo-Wiki location, with some variation in opinion between Gondor and Non-Gondor groups.

	Non-Gondor	Gondor
Forest	11	2
Shrub	13	17
Wetland	2	0

**Table 9:** An example of the land cover classes indicated a Geo-Wiki location, a high level of variation in opinion between Gondor and Non-Gondor groups.

	Non-Gondor	Gondor
Forest	1	1
Shrub	1	8
Grass	5	1
Crop	16	5
Barren	3	3

The existence of such variations has implications for the use of Geo-Wiki land cover data, which are currently being used to provide robust inputs to climate change models [26], for improved forest monitoring [43], to validate other datasets [51], to create hybrid global land cover datasets from existing (but uncertain) land cover data [13] and to support food security initiatives through agricultural land use mapping [52]. As yet none of these activities have sought to quantify or accounted for any variation between different groups of contributors and the uncertainties that such variations may have on the analytical outputs.

Consideration of inferential uncertainty is an important issue as the use of crowdsourced data in formal scientific analyses increases. Crowdsourced data are increasingly being used to replace data collected under formal experimental designs. Scientists are becoming more disengaged from the environments they study. Thus there is a need to consider the uncertainty associated with analysing such data. These issues were raised more than 20 years ago [53]. The context then was the relative nature of much geographical information and the associated uncertainties of using data that could be instantly downloaded via data portals rather than acquired through negotiation with a gatekeeper [54]. More recently these debates and the need to consider uncertainty have re-emerged in relation to Volunteered Geographical Information (VGI) and crowdsourced data [55]. This is especially relevant in the context of digital divides and their impact on the nature of the information that is contributed via citizen science activities, where there is an inherent potential for biases towards landscape concepts that are grounded in more developed countries using a particular and even biased set of landscape constructs and perceptions.

There are a number of areas for potential further work. First, there may be a need to refine how Geo-Wiki volunteers are recruited and whether this can be done in a more representative or even a targeted way. For example, data contributed by an individual who fails to meet some criteria may be excluded from analysis or perhaps their data re-interpreted via semantic translators. Second, Tables 7,8 and 9 suggest that considerable within group heterogeneity also exists, with likely impacts on data analyses. Further research is needed to explore the impacts of and to manage within group variation. This would help to determine whether local patterns of variation reflect within or between group differences / similarities for specific classes, in specific locations. One possibility is to take a mixed modelling approach to handle some of the independence issues relating to data being collected at different times, by different individuals, from different countries, from different images at different locations. Third, many of the Geo-Wiki datasets include measures of contributor confidence in the class labels for each point. These may provide a route to quantify uncertainties and label mismatches relative to the intended use of the data in analyses. Fourth, the ideal analysis would be one which compared a large number of classifications of the same locations by Expert / Non-Expert and Gondor / Non-Gondor users. Here a GW model was used to infer a spatial distribution of land cover from the Geo-Wiki point data, under the assumption that a degree of spatial autocorrelation exists. While much previous work has used similar approach there is a need to test this assumption at different scales of analysis. Additionally, understanding the sensitivity of the GW majority class assignment, particularly how these vary spatially, may provide insights into the differences observed between groups. Finally, Geo-wiki users register limited information about their background and experience. It would be useful to capture more structured information about the underlying semantics held by contributors relating to landscape processes. This could be extended to evaluate significant linguistic, epistemological and ontological differences among different groups, perhaps by allowing contributors to develop their own land cover categories. Identifying any cultural differences in this inductive way would allow contributors to classify data using their own knowledge base and language.

## Conclusions

The critical message arising from this research is that it is important to consider and test for potential variations in the way that landscape features are labelled and conceptualised by different groups of contributors when analysing crowdsourced data. It is not a question of the *veracity* of the citizen science data per se because that is dealt with by other research in this domain (see for example, [13, 28]). Rather, the issue is how to deal with and quantify the magnitude and direction of variations of crowdsourced data contributed by different groups. These include differences in the way that landscape features are described as well as the affordances and functions that they are associated with. Overcoming these issues is essential if crowdsourced data are to be robustly used in scientific analyses.

## Supporting Information

S1 Data The Geo-Wiki data used in this analysis, with attributes describing location (*Lon*, *Lat*), the allocated land cover class (*LC_Class*) and flags indicating whether the contributor was an Expert and was from Gondor. (CSV)

S1 Table The correspondence matrix of the land cover maps generated from data from All Contributors and those from Gondor. (DOCX)

S2 Table The correspondence matrix of the land cover maps generated from data from All Contributors and those from Non-Gondor. (DOCX)

S3 Table The correspondence matrix of the land cover maps generated from data from All Contributors and Expert contributors. (DOCX)

S4 Table The correspondence matrix of the land cover maps generated from data from All Contributors and Non-Expert contributors. (DOCX)
