# Supplementary material for: Crowdsourcing: It Matters Who the Crowd Are. The Impacts of between Group Variations in Recording Land Cover
Source: PLoS One. 2016 Jul 26;11(7):e0158329. doi: 10.1371/journal.pone.0158329 (PMC4961420; doi:10.1371/journal.pone.0158329)
Supplement: S1 Table — (DOCX) [file pone.0158329.s002.docx]

|  |  | Gondor | | | | | | | | |  |
| --- | --- | --- | --- | --- | --- | --- | --- | --- | --- | --- | --- |
|  |  | Forest | Shrub | Grass | Crop | Wetland | Urban | Snow | Barren | Water | Omission |
| All | Forest | 15936 | 1187 | 1510 | 147 | 100 | 64 | 92 | 346 | 489 | 0.20 |
|  | Shrub | 243 | 4405 | 96 | 45 | 0 | 15 | 3 | 84 | 22 | 0.10 |
|  | Grass | 1105 | 1113 | 5813 | 412 | 336 | 18 | 186 | 603 | 543 | 0.43 |
|  | Crop | 978 | 305 | 711 | 3566 | 34 | 26 | 1 | 194 | 20 | 0.39 |
|  | Wetland | 407 | 463 | 279 | 6 | 942 | 44 | 57 | 469 | 525 | 0.70 |
|  | Urban | 122 | 6 | 10 | 16 | 0 | 247 | 0 | 29 | 4 | 0.43 |
|  | Snow | 295 | 798 | 353 | 0 | 63 | 0 | 3071 | 235 | 368 | 0.41 |
|  | Barren | 528 | 1712 | 653 | 282 | 131 | 4 | 167 | 2474 | 765 | 0.63 |
|  | Water | 1120 | 639 | 376 | 106 | 35 | 41 | 213 | 386 | 4576 | 0.39 |
|  | Commission | 0.23 | 0.59 | 0.41 | 0.22 | 0.43 | 0.46 | 0.19 | 0.49 | 0.37 | 0.64 |

Table S1. The correspondence matrix of the land cover maps generated from data from All Contributors and those from Gondor.
