# Supplementary material for: Crowdsourcing: It Matters Who the Crowd Are. The Impacts of between Group Variations in Recording Land Cover
Source: PLoS One. 2016 Jul 26;11(7):e0158329. doi: 10.1371/journal.pone.0158329 (PMC4961420; doi:10.1371/journal.pone.0158329)
Supplement: S2 Table — (DOCX) [file pone.0158329.s003.docx]

|  |  | Non-Gondor | | | | | | | | |  |
| --- | --- | --- | --- | --- | --- | --- | --- | --- | --- | --- | --- |
|  |  | Forest | Shrub | Grass | Crop | Wetland | Urban | Snow | Barren | Water | Omission |
| All | Forest | 16552 | 275 | 1300 | 510 | 310 | 45 | 210 | 219 | 476 | 0.17 |
|  | Shrub | 421 | 1468 | 1131 | 127 | 335 | 3 | 330 | 370 | 718 | 0.70 |
|  | Grass | 808 | 66 | 7176 | 208 | 148 | 6 | 534 | 223 | 953 | 0.29 |
|  | Crop | 80 | 30 | 349 | 5299 | 17 | 16 | 0 | 6 | 39 | 0.09 |
|  | Wetland | 76 | 1 | 181 | 27 | 2786 | 0 | 37 | 55 | 30 | 0.13 |
|  | Urban | 37 | 4 | 3 | 9 | 9 | 350 | 0 | 1 | 21 | 0.19 |
|  | Snow | 120 | 0 | 68 | 1 | 14 | 0 | 4512 | 64 | 579 | 0.16 |
|  | Barren | 299 | 28 | 390 | 36 | 66 | 1 | 58 | 5970 | 20 | 0.13 |
|  | Water | 373 | 2 | 394 | 39 | 106 | 0 | 24 | 43 | 6859 | 0.13 |
|  | Commission | 0.12 | 0.22 | 0.35 | 0.15 | 0.27 | 0.17 | 0.21 | 0.14 | 0.29 | 0.79 |

Table S2. The correspondence matrix of the land cover maps generated from data from All Contributors and those from Non-Gondor.
