# Supplementary material for: Crowdsourcing: It Matters Who the Crowd Are. The Impacts of between Group Variations in Recording Land Cover
Source: PLoS One. 2016 Jul 26;11(7):e0158329. doi: 10.1371/journal.pone.0158329 (PMC4961420; doi:10.1371/journal.pone.0158329)
Supplement: S3 Table — (DOCX) [file pone.0158329.s004.docx]

|  |  | Expert | | | | | | | | |  |
| --- | --- | --- | --- | --- | --- | --- | --- | --- | --- | --- | --- |
|  |  | Forest | Shrub | Grass | Crop | Wetland | Urban | Snow | Barren | Water | Omission |
| All | Forest | 15475 | 969 | 1570 | 547 | 339 | 64 | 233 | 193 | 498 | 0.22 |
|  | Shrub | 355 | 2654 | 840 | 129 | 73 | 6 | 18 | 155 | 672 | 0.46 |
|  | Grass | 827 | 594 | 6059 | 221 | 268 | 11 | 720 | 446 | 974 | 0.40 |
|  | Crop | 498 | 59 | 471 | 4620 | 22 | 135 | 0 | 13 | 18 | 0.21 |
|  | Wetland | 122 | 122 | 87 | 8 | 2566 | 1 | 117 | 109 | 61 | 0.20 |
|  | Urban | 29 | 3 | 7 | 20 | 20 | 339 | 0 | 0 | 16 | 0.22 |
|  | Snow | 65 | 53 | 165 | 1 | 43 | 5 | 4080 | 353 | 590 | 0.24 |
|  | Barren | 500 | 653 | 513 | 42 | 112 | 3 | 166 | 4625 | 154 | 0.32 |
|  | Water | 553 | 486 | 826 | 313 | 570 | 4 | 92 | 67 | 4924 | 0.37 |
|  | Commission | 0.16 | 0.53 | 0.43 | 0.22 | 0.36 | 0.40 | 0.25 | 0.22 | 0.38 | 0.70 |

Table S3. The correspondence matrix of the land cover maps generated from data from All Contributors and Expert contributors.
