# Supplementary material for: Crowdsourcing: It Matters Who the Crowd Are. The Impacts of between Group Variations in Recording Land Cover
Source: PLoS One. 2016 Jul 26;11(7):e0158329. doi: 10.1371/journal.pone.0158329 (PMC4961420; doi:10.1371/journal.pone.0158329)
Supplement: S4 Table — (DOCX) [file pone.0158329.s005.docx]

|  |  | Non-Expert | | | | | | | | |  |
| --- | --- | --- | --- | --- | --- | --- | --- | --- | --- | --- | --- |
|  |  | Forest | Shrub | Grass | Crop | Wetland | Urban | Snow | Barren | Water | Omission |
| All | Forest | 16944 | 407 | 863 | 503 | 141 | 43 | 98 | 162 | 724 | 0.15 |
|  | Shrub | 374 | 3172 | 588 | 31 | 62 | 4 | 69 | 304 | 308 | 0.35 |
|  | Grass | 1247 | 348 | 6632 | 271 | 374 | 10 | 123 | 758 | 375 | 0.35 |
|  | Crop | 741 | 149 | 287 | 4431 | 4 | 40 | 3 | 171 | 9 | 0.24 |
|  | Wetland | 424 | 89 | 451 | 25 | 1284 | 29 | 196 | 511 | 184 | 0.60 |
|  | Urban | 58 | 1 | 6 | 9 | 4 | 340 | 1 | 4 | 11 | 0.22 |
|  | Snow | 264 | 158 | 190 | 0 | 152 | 1 | 3899 | 499 | 58 | 0.25 |
|  | Barren | 159 | 352 | 516 | 24 | 126 | 0 | 431 | 5070 | 190 | 0.26 |
|  | Water | 716 | 570 | 172 | 9 | 49 | 15 | 95 | 482 | 5406 | 0.28 |
|  | Commission | 0.19 | 0.40 | 0.32 | 0.16 | 0.42 | 0.29 | 0.21 | 0.36 | 0.26 | 0.74 |

Table S4. The correspondence matrix of the land cover maps generated from data from All Contributors and Non-Expert contributors.
